# Supplementary figures and images for: Germplasm Resources of Oaks (Quercus L.) in China: Utilization and Prospects
Source: Biology (Basel). 2022 Dec 31;12(1):76. doi: 10.3390/biology12010076 (PMC9855944; doi:10.3390/biology12010076)

# *Quercus acutissima*

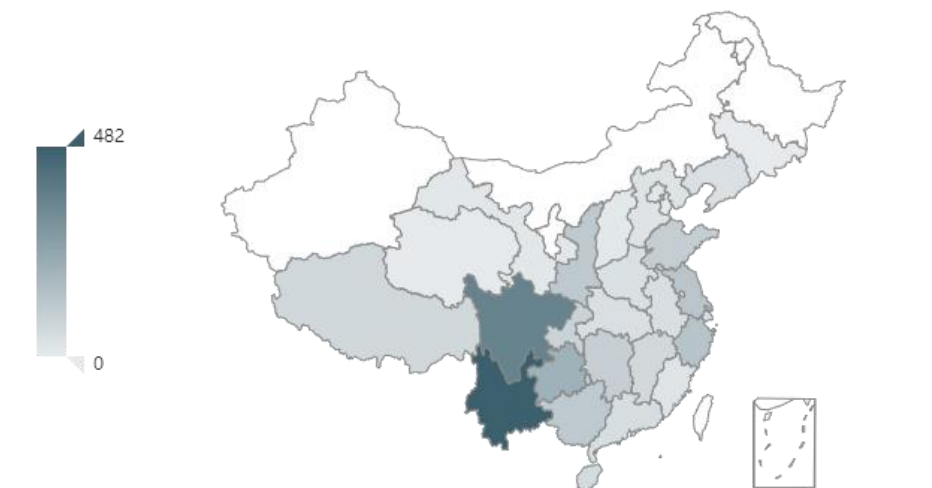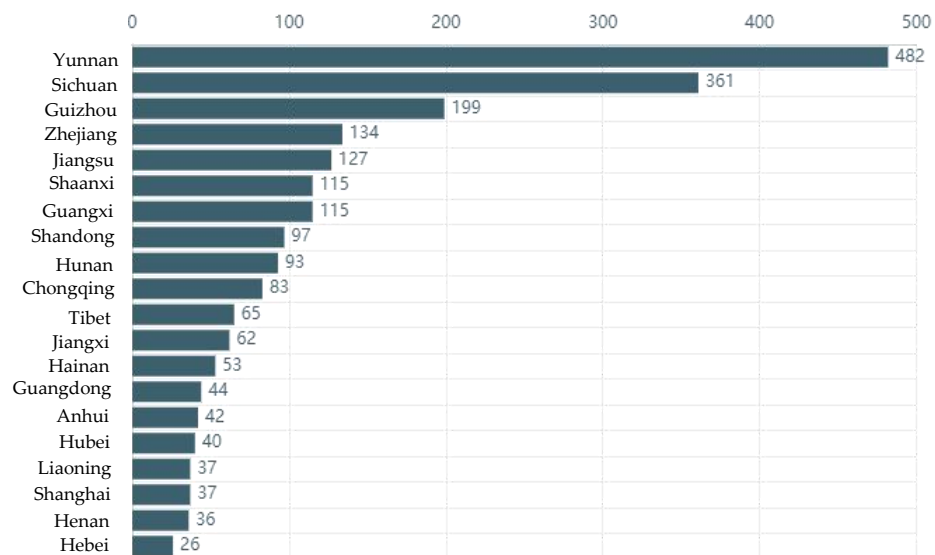

# *Quercus dentata*

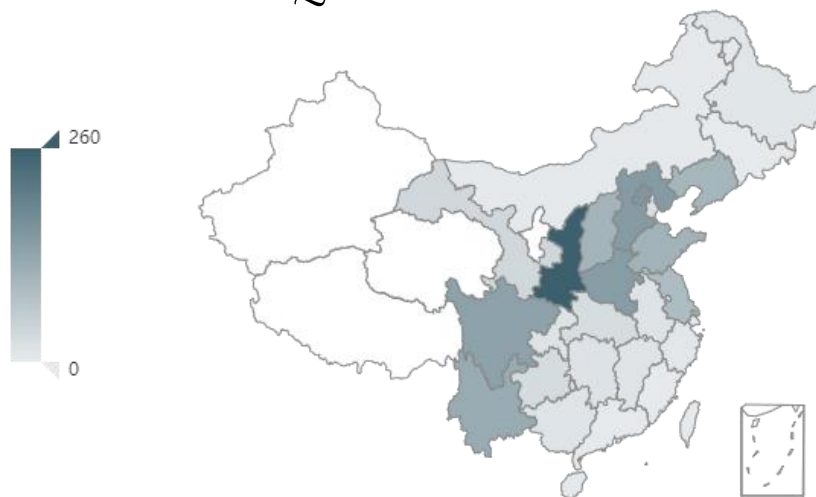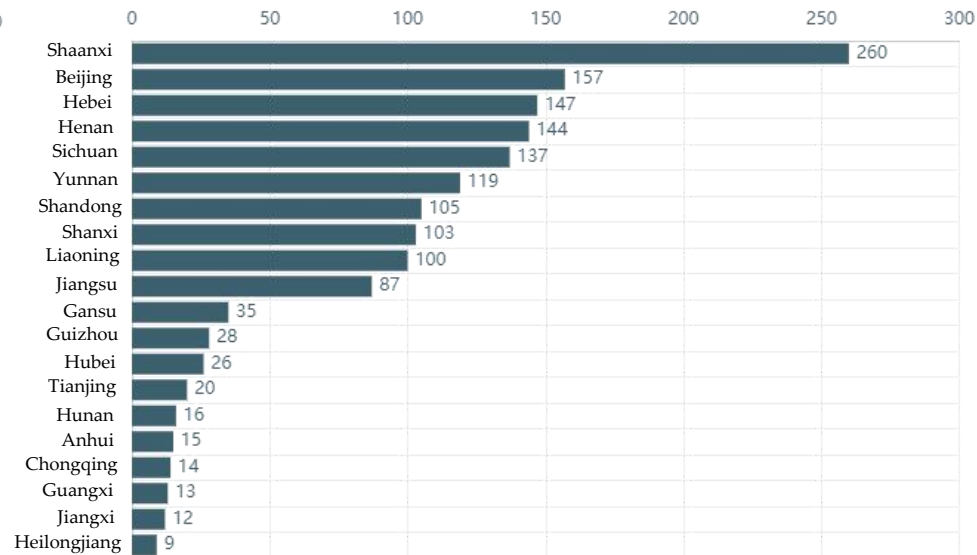

Supplement Figure S1

Top 20 provinces of samples collected

Supplement: Supplementary file 1 [file biology-12-00076-s001.zip › biology-2091803-supplementary.pdf]
